# Supplementary material for: Antihypertensive treatment and risk of cardiovascular mortality in patients with chronic kidney disease diagnosed based on the presence of proteinuria and renal function: A large longitudinal study in Japan
Source: PLoS One. 2019 Dec 4;14(12):e0225812. doi: 10.1371/journal.pone.0225812 (PMC6892527; doi:10.1371/journal.pone.0225812)
Supplement: Attachment — Submitted filename: comment to authors PONE-D-19-26343.docx [file pone.0225812.s001.docx]

Nagai et al reported the association between antihypertensive treatment and CVD mortality in patients with CKD who received annual health checkups. This study is a longitudinal general-population cohort with a large number of subject and showed the clear results of an increased mortality in those with hypertension or with hypertensive treatment, thus the data is of clinical importance. However, this manuscript has some major problems.

1)In Fig2, authors showed hazard ratio of hypertensive group or treated groups, referencing untreated normal blood pressure. Authors conclusion is the effectiveness of BP reduction in preventing CVD in CKD patients, however, in this manuscript, it is unclear whether hazard ration between untreated hypertensive people, treated normal BP people and treated hypertensive people in CKD is statistically different. Authors should clearly show this point.

2)In Fig2, authors showed hazard ration of CVD mortality in non-CKD people. These data revealed the CVD risk of hypertension, however did not show the effectiveness of hypertensive treatment for the prevention of CVD risk in non-CKD people. Is BP control not effective for the CVD reduction in non-CKD hypertensive subjects in this study? Authors should describe the results although this manuscript targets CKD patients.

3) Table3 showed CVD mortality in those with CKD and without CKD. In conclusions, authors described “…CVD mortality risk seems to be higher in CKD patients than in non-CKD patients.”. Authors should show statistical significance.
