# Supplementary material for: Antihypertensive treatment and risk of cardiovascular mortality in patients with chronic kidney disease diagnosed based on the presence of proteinuria and renal function: A large longitudinal study in Japan
Source: PLoS One. 2019 Dec 4;14(12):e0225812. doi: 10.1371/journal.pone.0225812 (PMC6892527; doi:10.1371/journal.pone.0225812)
Supplement: Attachment — Submitted filename: RtoRs-2019-10-POne-r1.docx [file pone.0225812.s002.docx]

Re: PONE-D-19-26343, Antihypertensive treatment and risk of cardiovascular mortality in patients with chronic kidney disease diagnosed based on the presence of proteinuria and renal function: A large longitudinal study in Japan

**Response to reviewers:**

**Reviewer #1:**

[Major points]

1-1) Since CVD mortality in CKD patients with normotensive/hypertension treated category (0.35%) was lower than that with hypertensive/untreated category (0.39%), the authors interpreted that the findings support treatment of hypertension in CKD patients to reduce CVD death. However, this is an observational study and cause/result relationship and speculation for prospective interventional benefits cannot be obtained, since patients in these 2 categories may have different basal backgrounds. For instance, patients with untreated hypertension may have lower socioeconomical conditions and may be less concerned about their health. Normotensive patients under hypertension treatment may have a better chance to be diagnosed other disorders during their periodical hospital visit and receive better care. There is no guarantee that untreated subjects remain untreated for 4 years.

Thank you for your comment and we recognize this observational study cannot show the interventional effect of BP control on CVD mortality and hard to discuss magnitude of HRs due to unadjustable factors among hypertension treatment categories before baseline year such as socioeconomical status and hospital visiting. Therefore, we would like to add limitation in the second last paragraph of the discussion and deleted description to compare HRs between two hypertension treatment categories other than reference group.

1-2) CVD mortality in non-CKD subjects were 0.21% for normotensive/hypertension treated category and 0.17% for hypertensive/untreated category (Table 3). Furthermore, CVD mortality in overall population (including CKD and non-CKD) were 0.27% for normotensive/treated category and 0.22% for hypertensive/untreated category (by reviewer's calculation from Table 3). These findings prove that authors' logic is inconsistent to established effects of hypertension treatment to reduce CVD death in CKD and non-CKD subjects.

Thank you for your thoughtful comment. Related to your 1^st^ major point, we recognized that it is hazardous to compare HRs each other between two hypertension treatment categories other than reference group (i.e. untreated normal BP). We would like to correct the conclusions from “effect of hypertension treatment on mortality” into “current demographics of hypertension treatment in Japan”. Please approve to mix up discussion and conclusion sections in order to keep context fluency (PLoS One editorial approves it by the author instruction).

1-3) As the authors described, previous studies reported CVD mortality among subjects with reduced renal function. The present study is distinct in that not only reduced renal function but also proteinuria was analyzed.

Thank you for your suggestion, we highlighted strong point of our study to deal with proteinuria, in the fourth paragraph of the revised discussion.

1-4) Median follow up period should be described in the abstract.

Thank you for your comment. We added follow-up period in the revised abstract and method section.

1-5) CVD mortality in CKD and non-CKD subjects should be described in the abstract.

Thank you for your comment. We added description regarding CVD mortality in the revised abstract.

[Minor points]

2-1) A term "hypertensive category" is often used in the manuscript but it implies the extent of hypertension: for instance, 140, 160 and 180 mmHg. Other term, such as hypertension treatment categories, should be appropriate.

Thank you so much for your suggestion and we agree with you. We used “hypertension treatment category” throughout this manuscript.

2-2) Line 138: >5 years should be replaced by 5.5 years.

Thank you for comment. Please make sure it in the revised version.

**Reviewer #2:**

1) In Fig2, authors showed hazard ratio of hypertensive group or treated groups, referencing untreated normal blood pressure. Authors conclusion is the effectiveness of BP reduction in preventing CVD in CKD patients, however, in this manuscript, it is unclear whether hazard ration between untreated hypertensive people, treated normal BP people and treated hypertensive people in CKD is statistically different. Authors should clearly show this point.

Thank you for your thoughtful comment. As another reviewer and you pointed out, we recognized that it is hard to compare HRs each other between two hypertension treatment categories (ie. untreated hypertension vs treated normal BP), other than reference group (untreated normal BP). We would like to correct the conclusions from “effect of hypertension treatment” into “current demographics of hypertension treatment in Japan”. Please approve to mix up discussion and conclusion sections in order to keep context fluency (PLoS One editorial approves it by the author instruction).

2) In Fig2, authors showed hazard ration of CVD mortality in non-CKD people. These data revealed the CVD risk of hypertension, however did not show the effectiveness of hypertensive treatment for the prevention of CVD risk in non-CKD people. Is BP control not effective for the CVD reduction in non-CKD hypertensive subjects in this study? Authors should describe the results although this manuscript targets CKD patients.

Related to your 1^st^ point, we should not have compared HRs between untreated hypertension and treated normal BP, among CKD subjects as well as among non-CKD. We appropriately deleted description regarding effectiveness of BP control and just touched on the previous works in CKD cohort and implication of marked CVD mortality risk in patients with positive proteinuria in the fourth paragraph of the revised manuscript.

3) Table3 showed CVD mortality in those with CKD and without CKD. In conclusions, authors described "...CVD mortality risk seems to be higher in CKD patients than in non-CKD patients.". Authors should show statistical significance.

Thank you for your thoughtful comment. Related to your former comments, we revised conclusion, because it is difficult to compare HRs among hypertension treatment categories. Please make sure the last paragraph of the revised discussion section.

We would like to thank all the reviewers again.

Kunihiro Yamagata MD., PhD.

Department of Nephrology, Faculty of Medicine, University of Tsukuba,

1-1-1 Ten-noudai,

Tsukuba, Ibaraki, 305-8575, Japan

Tel.: +81-298-53-3202

Fax: +81-298-53-3202

E-mail: [k-yamaga@md.tsukuba.ac.jp](mailto:k-yamaga@md.tsukuba.ac.jp)
